# Analysis of Copper Concentration in Human Serum by Application of Total Reflection X-ray Fluorescence Method

**DOI:** 10.1007/s12011-013-9884-4

**Published:** 2014-02-27

**Authors:** Aldona Kubala-Kukuś, Dariusz Banaś, Janusz Braziewicz, Urszula Majewska, Marek Pajek, Jolanta Wudarczyk-Moćko, Grażyna Antczak, Beata Borkowska, Stanisław Góźdź, Jolanta Smok-Kalwat

**Affiliations:** 1 Institute of Physics, Jan Kochanowski University, Świȩtokrzyska 15, 25-406 Kielce, Poland; 2 Holycross Cancer Center, Artwińskiego 3, 25-734 Kielce, Poland; 3 Institute of Public Health, Jan Kochanowski University, IX Wieków Kielc 19, 25-317 Kielce, Poland

**Keywords:** Copper in human serum, TXRF method, Chemotherapy, Log-normal distribution, Log-stable distribution, Copper reference level

## Abstract

The chemotherapy and photon radiotherapy are the most often applied methods in treatment of the cancer diseases because of their effectiveness and high cure rates. Apart from eligible destruction of the tumour, one of the side effects of these treatment methods is possible modification of main and trace element concentration in different human tissues and fluids. In this paper, the copper (Cu) level in human serum was determined by total reflection X-ray fluorescence method in 142 chemotherapy patients and in 44 healthy persons being a control group. The Cu concentration in the chemotherapy group was found to be on the level 1.78 ± 0.909 mg/L, while in the control group, it was 1.08 ± 0.551 mg/L. Performed measurements allowed for calculation of the parameters of copper concentration distribution (mean value, standard deviation, median) for both analysed groups. The theoretical nature of the concentration distribution was tested and found as a log-normal distribution (control group) and a log-stable distribution (chemotherapy group). The copper concentration distributions for both studied group were statistically compared using Kolmogorov-Smirnov test, and the conclusion was that the distributions are statistically different. Serum Cu levels were significantly higher in the chemotherapy group than in the control group. Taking into account the results for the control group, the copper concentration reference quantile ranges in human serum were obtained. The values of the mean, median and other quantiles determined in this case can be applied in two-group comparison studies. The obtained results can be used as a diagnostic tool for chemotherapy patients.

## Introduction

The contents of trace elements in biomedical samples are of great interest for biology, medicine and environmental sciences. In our laboratory, different types of biomedical samples, e.g. urine, serum, blood, breast, foetal membranes and placenta samples have been studied in recent years [1]. Studies on the concentration of trace elements in biomedical samples have been performed by application of one of the most known techniques, namely the total reflection X-ray fluorescence (TXRF) method [2]. This method allows fast and accurate, simultaneous analysis of all elements heavier than aluminium (for measurements in air) and is routinely used in medical applications, in particular, to study on the content of heavy metals in different samples of medical interest [1, 3–5]. Our investigations had different motivations but were mainly focused on the development of new possibilities of cancer diagnosis and therapy monitoring as well as investigation of people’s health related to different aspects of environmental pollution. Performed measurements yielded rather systematic data on the concentration distributions of trace elements in biomedical samples, thus allowing a formulation of general conclusions related to the nature of observed concentration distributions as well as the role of detection limits and resulting nondetects, which are of practical importance for the application of the total reflection X-ray fluorescence analysis in trace element studies. One of the routine analyses performed in our laboratory is the determination of element concentration in the serum of chemotherapy or/and radiotherapy patients in Holycross Cancer Center (Kielce, Poland). The photon radiotherapy and chemotherapy are the most often applied methods in the treatment of cancer diseases. Knowledge on the biology of normal cells and cancer cells allowing the understanding on the growing processes of the cancer tissue, together with the knowledge on the interaction of gamma rays with the matter, gives the possibility of effective fight with cancer. The reaction which occurs between the gamma rays and human body and the influence of the chemotherapy on the organism leads to metabolic changes. One of the effects of the discussed treatment methods is modification of main and trace element concentration in different human tissues and fluids [6, 7]. It has been found that copper concentration in serum correlates with tumour incidence and burden, malignant progression and recurrence in many different human cancers [6–8]. For example, an increased level of copper in the serum is observed for Hodgkin’s disease, and changes in copper concentration in human serum are observed during the chemotherapy process [7, 8]. The observation of these modification before, during and after radiotherapy and chemotherapy, can give information about the effectiveness of the treatment process. The main aim of this presented study is a wide and systematic analysis of element concentration in different human biological materials (serum, hair, tissue) of radiotherapy and chemotherapy patients, and application of obtained results in formulating a general observation which can be used as a tool for monitoring the treatment process. The study is performed with the approval of the Bioethics Committee at Holycross Medical Chamber. Under the proposed research programme, which is the first stage of the described long-term study, we have started monitoring the copper concentration in human serum of patients with malignant tumour during chemotherapy. Copper is an essential trace element with many physiological functions. It affects the activity of many enzymes, which are essential for cellular respiration, defence against free radicals, formation of connective tissue and for iron metabolism [9]. In the context of the cancer diseases, the role of cooper in tumour angiogenesis has been demonstrated, and the reduction of the copper level in cancer patients has become one of the approaches in antiangiogenic treatment [9]. Taking into account that a common problem in biomedical studies is the comparison of trace element contents in two, usually differently selected, populations or control group, the performed analysis was also supplemented by systematic studies on copper level in the group of healthy persons. In the case of the latter group, the main aim was determination of the reference level of copper concentration in human serum, understood as a mean value or a quantile range for different confidence levels. These values can be next used in cancer research programme. In this presented paper, the experimental setup of the TXRF method and the calibration procedure will be briefly discussed. Next, the groups of the samples and the sample preparation procedure will be described. The results of copper concentration analysis in serum for the control group and chemotherapy group will be presented together with two-group comparison. In performed systematic investigations of copper concentration in human serum samples by using the total reflection X-ray fluorescence method, taking into account a large number of analysed samples, it will be possible to describe the nature of these distributions, and also several important methodological aspects related to copper concentration distributions can be addressed.

## Material and Methods

### Method of Analysis

**Fig. 1:**
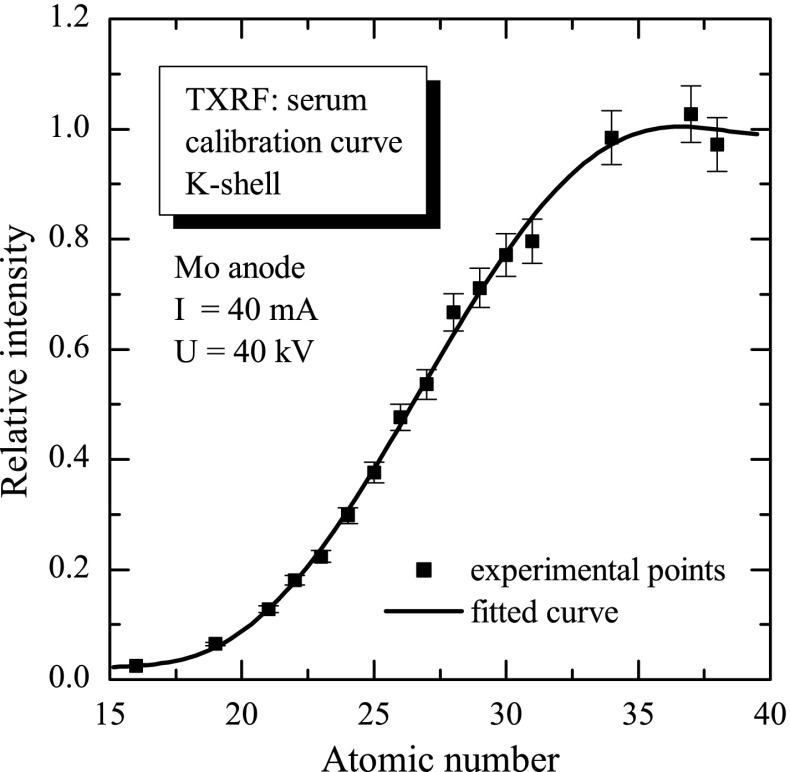
The calibration curve of TXRF method for the elements analysed by the identification of the characteristic K-shell X-ray emitted from the sample. The calibration curve allows for the quantitative analysis of the analysed samples

**Fig. 2:**
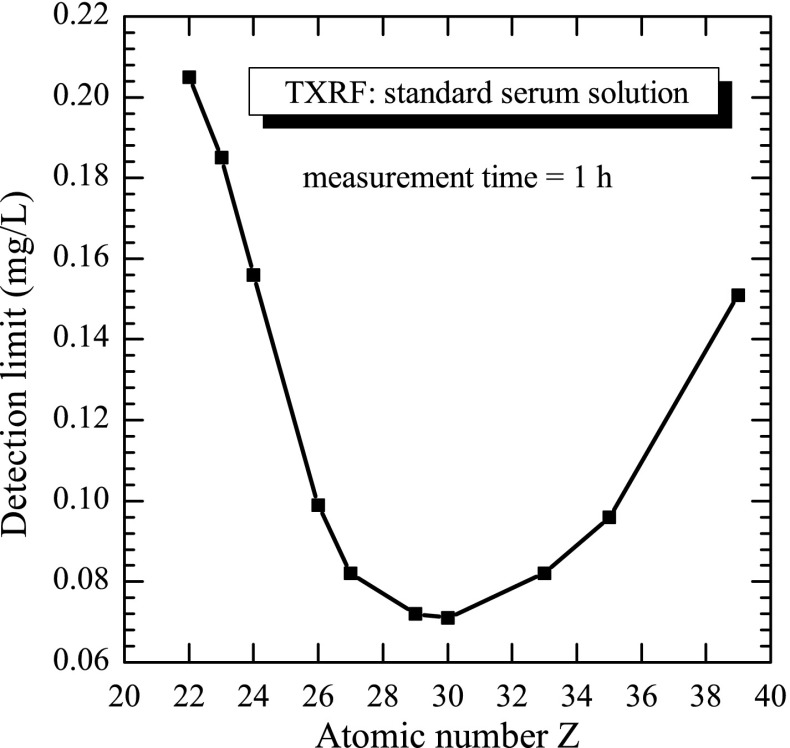
The dependence of the detection limit on the atomic number of the analysed element obtained for use in the presented study on TXRF method setup. The values of the level of detection limit were obtained for human serum sample for the 1 h measurement time. For copper (atomic number *Z* = 29), the detection limit of the TXRF technique is on the level of 0.07 mg/L

In the present study, the TXRF method was used to determine the concentration of copper in human serum. This method is a modification of the X-ray fluorescence method (XRF) [10, 11] with special experimental geometry. In classical XRF technique, X-ray is directed on the analysed samples at the angle close to 45° and a detector registering the characteristic X-ray emitted from the sample is mounted in the geometry of 90° with respect to the primary beam of X-ray photons (mainly for reducing the detection of X-ray scattering). The main idea of the experimental geometry modification in TXRF technique is that incoming X-ray is directed on a flat carrier disc, usually polished quartz (or silicon wafer), on which the analysed sample is deposited, at a small angle, being below the critical angle of the X-ray total external reflection. The value of critical angle depends on the X-ray energy and on the carrier disc material, and for quartz and energy 17.44 keV (Mo-K*α* line) is equal to 0.1°. Next, the incoming X-rays are totally reflected from the carrier disc, and as the result, the primary beam penetrates the sample only on the depth of a few nanometers. The scattering processes are reduced, and the background in the analysed X-ray spectrum is much lower. Additionally, the characteristic X-ray detector is mounted close (∼3–4 mm) to the analysed sample (still in geometry 90°). Consequently, the detection limit is improved about factor 10^3^. In the presented study, the characteristic X-rays were excited in the samples by a collimated beam of photons from 2-kW Siemens Mo-anode X-ray tube operated at 40 kV with an electron current of 40 mA. The total reflection X-ray measurements were performed using the TXRF attachment module [2] developed at Atominstitut, Vienna. The primary X-rays from the tube were first collimated to a size of 50 μm × 10 mm. Next, the X-rays were directed onto a reflector which, as a results of the X-ray’s total external reflection phenomenon, cuts off the high-energy X-rays just above the Mo-K *α* line. The reflected X-ray beam was formed by the collimator additionally mounted in our experimental setup for improving the detection limit of the TXRF method by a reduction of the X-ray’s scattering [12]. The primary beam, formed in such way, was next directed onto the studied sample. The fluorescence characteristic of X-rays from the samples was detected by a Si(Li) detector having an energy resolution of 170 eV at 6.4 keV. The calibration of the instrument was performed using both ICP Multi-Elemental Merck Standard Solution IV (Ag, Al, B, Ba, Bi, Ca, Cd, Co, Cr, cu, Fe, Ga, In, K, Li, Mg, Mn, Na, Ni, Pb, Sr, Tl, Zn) and mono-element (Ag, As, Ca, Cd, Cl, K, P, Rb, S, Sc, Se, Ti, W) Merck standard calibrating solutions. The initial concentration of each solution was 1,000 mg/L. The calibration procedure and, consequently, the routine measurements were performed with application of yttrium as an internal standard. The calibration measurements were next applied in the calculation of the calibration curve, as presented in Fig. 1 for the elements analysed by the identification of the characteristic K-shell X-ray lines emitted from the sample. The calibration curve allows for the quantitative analysis of the analysed samples. For the XRF/TXRF methods, the detection limit for the determination of the concentration of a given element depends on the intensity of characteristic X-ray line of interest as well as the level of background intensity and measurement time. More precisely, a concentration of trace element can be determined by the X-ray fluorescence only when the total number of counts in the corresponding X-ray line, *N*_peak_, exceeds in a statistically significant way the corresponding background level, *N*_bkg_, in the measured X-ray spectrum. Usually, one assumes that the concentration detection limit *C*_*DL*_ is defined by the “three standard deviation rule,” which, for the counting process, reads as follows: $N_{\rm{peak}}\geq{3\sqrt{N_{\rm{bkg}}}}$ [2]. Both the peak and background intensities, *N*_peak_ and *N*_bkg_, respectively, can be routinely obtained by using the available X-ray spectra fitting software, and consequently, the values of measured concentrations C and detection limits *C*_DL_ can be calculated from the calibration sensitivity curve of the method. The detection limit for the different elements in a human serum sample for the 1-h measurement is presented in Fig. 2. For copper (atomic number *Z* = 29), the detection limit of the TXRF technique for our experimental setup is on the level of 0.07 mg/L, which is much lower than the physiological values observed for copper concentration in human serum. Additionally, our elemental analysis characterizes the accuracy on the level of 15 % and repeatability of about 5 %.

### Human Serum Samples

**Fig. 3:**
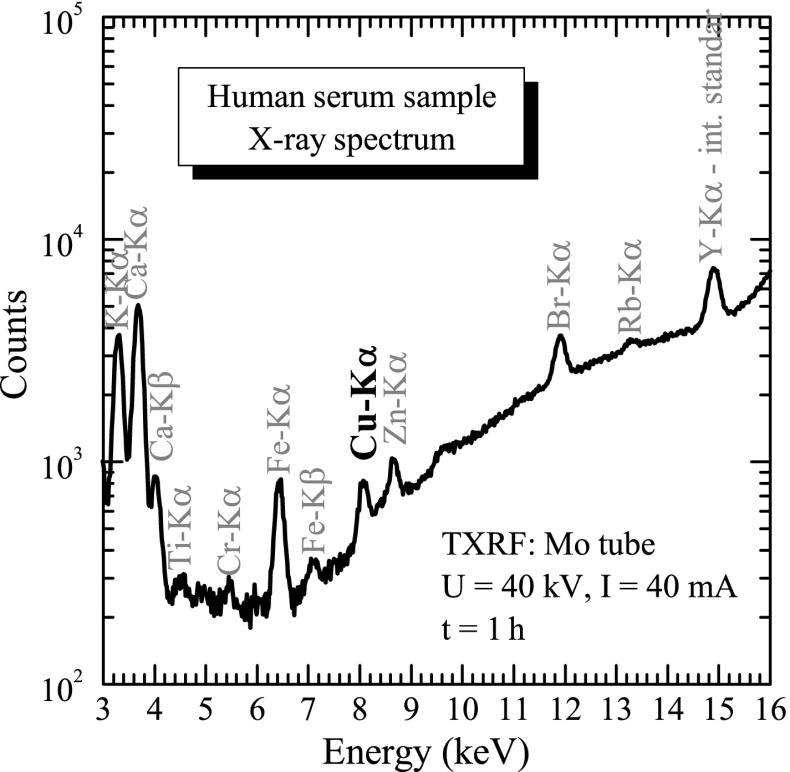
A typical TXRF spectrum of a human serum sample excited by the primary X-rays emitted from a Mo-anode X-ray tube. The yttrium K *α* X-ray line is from the internal standard added for the calibration purpose. The copper atoms are identified in the measured spectrum by their characteristic K *α* X-rays (marked in *bold*). In the measured X-ray spectrum, also the characteristic X-rays of other elements are observed

The total reflection X-ray fluorescence method in the presented research programme was applied for the determination of the copper concentration of two groups of human serum samples. The first group of samples, called control group (CG), consisted of the serum samples of 44 healthy persons between 18 and 45 years of age who donated blood voluntarily, being employees of Holycross Cancer Center (HCC) in Kielce, Poland, undergoing periodic examination. The second group of the human serum samples, called further chemotherapy group (CHG), was the serum of 142 patients with malignant tumour who were at the beginning of chemotherapy process. The main aim of the analysis was the determination of the copper concentration in serum. This information is used by the doctors in diagnostic process. The samples of blood were taken early in the morning from an elbow vein of each patient after 12 h without food and put into sterile tubes (disposable vacuum tubes BD Vacutainer) with blue stopper, for the analysis of trace elements, made of transparent plastic—polyethylene terephthalate (PET)—containing a clotting activator. The time of transport of the blood to the laboratory is up to 4 h, and the temperature upon transport is 15–25 °C. Next, the serum was prepared from each blood sample by centrifugation. In the centrifugation process, the laboratory centrifuge MPW-350e was used, with time of centrifugation of 10 min and centrifugation rate of 1,669*g* (RCF). After centrifugation, serum sample was transferred to an Eppendorf and frozen with a temperature of about −20 °C. A determined amount of serum (usually 0.5 mL, but in some cases 0.2 mL due to insufficient amount of serum) of each patient of the control group was separately mineralized in a closed glass bottle with 1 mL of high-purity HNO_3_ acid, 0.5 mL of H_2_ *O*_2_ (30 %), and 300 μL of water solution of Y(NO_3_)_2_ as an internal standard. The serum samples of the chemotherapy group were separately mineralized in a closed glass bottle with 1 mL of high-purity HNO_3_ acid, 0.5 mL of H_2_ *O*_2_ (30 %), and 100 μL of water solution of Y(NO_3_)_2_ as an internal standard. Next, in both cases, 5 μL of solution was pipetted into quartz carrier disc, and this drop was dried in infrared. The sample preparation method was checked experimentally to determine possible volatility losses, which could occur for some more volatile elements. In fact, such losses were negligible for the copper concentration, assuming the above-described sample mineralization procedures. The prepared sample was next analysed using TXRF methods. The X-ray spectra were collected for a 1-h time for each sample. The measurement time was 1 h mainly due to the observation that copper concentration can be on a quite low level (∼0.2 mg/L), while the background observation in serum sample spectra is relatively high. Additionally, in the future, it can be possible to also analyse other elements from the measured spectra, so the 1-h measurement time is a compromise between the sample throughput and possibility of other element detection. A typical example of the TXRF X-ray spectrum measured for the human serum is presented in Fig. 3. In the X-ray spectra, the peak of copper was identified. Additionally, K- and L-lines of the other elements (for example, K, Ca, Ti, Cr, Fe, Zn, Br, Rb, Pb) were also observed. In the first step of the discussed research programme, the level of copper in the human serum samples was taken into account, and the concentration of this element was calculated using calibration curve. The experimental methodological uncertainties of measured concentrations were usually within 15 %.

## Results and Discussion

**Fig. 4:**
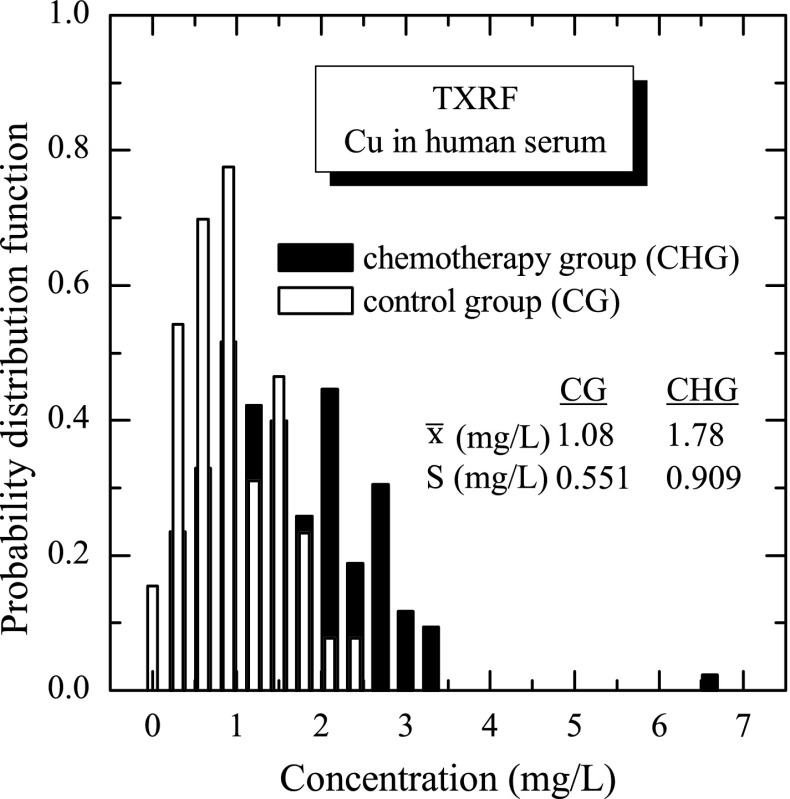
The histograms of the copper concentration distributions measured by TXRF method in two groups of human serum samples, namely in control and chemotherapy patients groups. The estimated mean values (*x̄*) and standard deviations (*S*) are shown in the figure

**Fig. 5:**
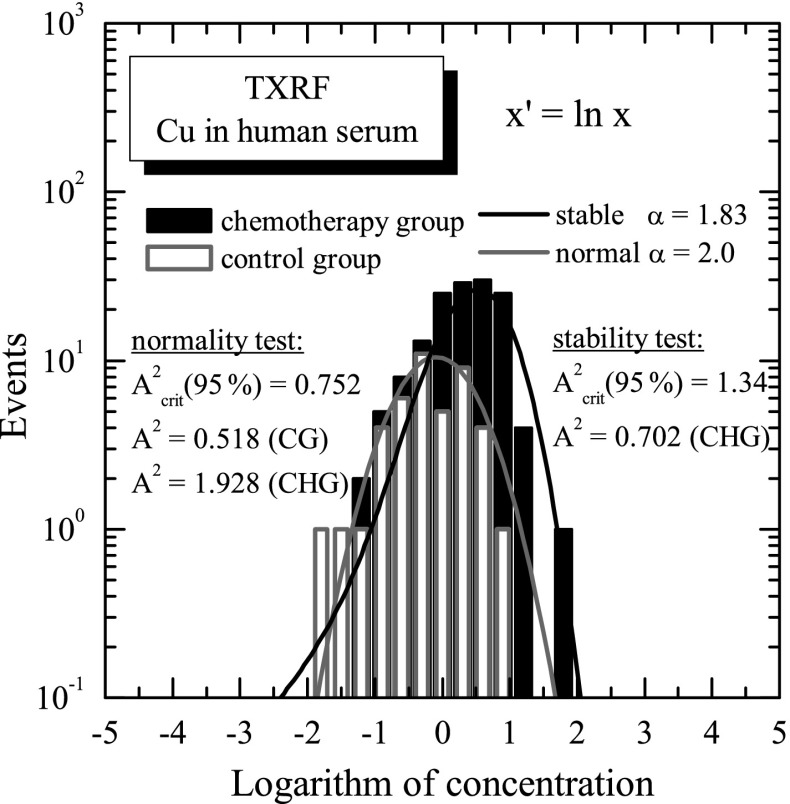
The histograms of the logarithmically transformed (*x*′ = *lnx*) copper concentration distributions measured by the TXRF method in two groups of human serum samples: in control and chemotherapy patients groups. The histograms are presented with fitted normal distribution (*control group*) and symmetric *α*-stable distribution S_*α*_(μ, *σ*,0) (*chemotherapy group*). The case of the normal distribution corresponds to the stability index *α* = 2.0. The Anderson-Darling test supported, at the confidence level of 95 %, a normality of the transformed concentration distribution for the control group and stability (*α* = 1.83) for the chemotherapy group

The results were carefully analysed to investigate the concentration distribution of copper in the studied two groups of human serum samples. The parameters of concentration distributions, i.e. mean value, standard deviation and median, were calculated both for control and chemotherapy groups.The calculated values are summarized in Table 1. In the table, also the numbers of studied samples and range of the measured copper concentration are presented. It was observed that the group of chemotherapy patients is characterized by higher values of all parameters compared to the control group. Moreover, the range of the observed copper concentrations, defined as the range from the lowest to the highest measured concentration, is much wider in the chemotherapy group. Also extremely high concentrations, sometimes called “outliers,” are observed in this group. The variation coefficient for both groups is almost on the same level. The mean value of the copper concentration (1.08 mg/L) in human serum observed for the control group agrees with the results of other authors [6]. Compared with the mean value, a relatively high value of the standard deviation of the copper concentration distribution is, for this group, a result of physiological reason and biological variation in the individual person, as influenced by many different factors (sex, age, diet, environmental pollution, etc). Analysing the chemotherapy group, separately for males (77 samples) and females (65 samples), very similar values of copper concentration were obtained for male and female groups, namely mean 1.777 and 1.775 mg/L; median 1.722 and 1.652 mg/L; and standard deviation 0.951 and 0.864 mg/L, respectively. Taking into account that these results and the fact that no statistical significant differences were observed between means, the male and female groups were not distinguished and were analysed as one group. The distributions of copper concentration in analysed human serum samples were graphically presented in Fig. 4. As it is shown in the figure, the histograms were found to be strongly asymmetric and long-tailed, as it was similarly observed for other medical samples in our earlier studies [1]. It is worth to mention here that such asymmetric concentration distributions of trace element in medical samples are well described by the log-normal distribution or more general log-stable distribution [13]. The log-stable character of the measured concentration distributions of trace elements was evidenced by demonstrating that the logarithmically transformed concentrations (*x*′ = ln*x*) are described by the *α*-stable distribution S_*α*_(μ, *σ*, *β*) [13], where *α* is the index of stability; *μ* and *σ* are the location and scale parameters, respectively; and *β* is asymmetry parameter. Taking into account that the *α*-stable distribution for *α* = 2 is the Gaussian (normal) distribution, the theoretical model yields in this case the log-normal distribution of concentrations. For lower values 0 < *α* < 2, the concentrations have the log-stable distributions having power-like tails, in contrast to the log-normal distribution characterized by the exponential tails. In fact, the “outliers,” which are observed in the measured distributions, can in fact manifest the log-stable nature of the studied concentration distributions. The results of the logarithmic transformation of the measured concentration distributions of copper in the control and chemotherapy groups are shown in Fig. 5. The *α*-stable distributions S_*α*_(μ, *σ*, *β*) were fitted to the logarithmically transformed (*x*′ = ln*x*) concentrations using the STABLE code developed by Nolan [14, 15]. In this fitting, only the symmetric stable distributions S_*α*_(μ, *σ*,0) were considered, which were a sufficient approximation tested separately. Figure 5 shows the fitted values of the stability index *α* for the copper concentration distribution in the control (CG) and chemotherapy (CHG) groups. The concentrations of copper in the control group were found to be well described by the log-normal model (*α*= 2.0), while the copper concentrations in the chemotherapy group could be only well reproduced by the log-stable model with stability index *α* = 1.83. This observation was supported by the Anderson-Darling *A* ^2^ test [16], known as very sensitive for the tails of distribution, used as a “goodness-of-fit” test. The *A* ^2^ test was applied both for testing the normality of the copper distribution in the control group and for testing the normality and stability of the copper distribution in the chemotherapy group. Finally, the Anderson-Darling test supported, at 95 % confidence level, that the measured concentration distribution of copper is log-normal (*α* = 2.0) for the control group while log-stable (*α* = 1.83) for the chemotherapy group. The main interpretation of the log-stability of the copper serum concentration distribution in the chemotherapy group is a fact that in the log-stable distributions, extremely high or extremely low values of concentration can be observed with higher probability, compared with log-normal distributions. It is known that the copper level in the serum of tumour patients is a result of many unexpected and complex factors and processes [18]. Copper stimulates the proliferation and migration of endothelial cells and is required for the secretion of several angiogenic factors by tumour cells. Serum copper levels are upregulated in many human tumours and correlate with tumour burden and prognosis. Tumours may exploit the role of copper by upregulating the expression of copper-carrying proteins and pathways [18]. These processes can result in extremely high values of copper serum concentration, which confirms the presence of the log-stable nature of the concentration distribution. It is worth emphasizing that copper chelators reduce tumour growth and microvascular density, and synergy between copper chelation and other treatment modalities such as chemotherapy and radiotherapy is observed [18]. In sum, on the one hand, the information about the kind of copper concentration distribution can model a process of copper accumulation in the serum, but on the other hand, this information is a fact of practical importance for further statistical data analysis, for example, for a two-group comparison.

**Table 1:** Mean values, medians and standard deviations of copper concentrations in the human serum samples for two studied groups, namely for control and chemotherapy groups

Group (Number of samples)	Parameters (mg/L)			
	Mean value	Median	Standard deviation	Range (min ÷ max)
Control (44)	1.08	0.97	0.551	0.213 ÷ 2.50
Chemotherapy (142)	1.78	1.68	0.909	0.397 ÷ 6.64

Also the numbers of studied samples and the range of the measured copper concentration are presented

### Comparative Analysis

**Fig. 6:**
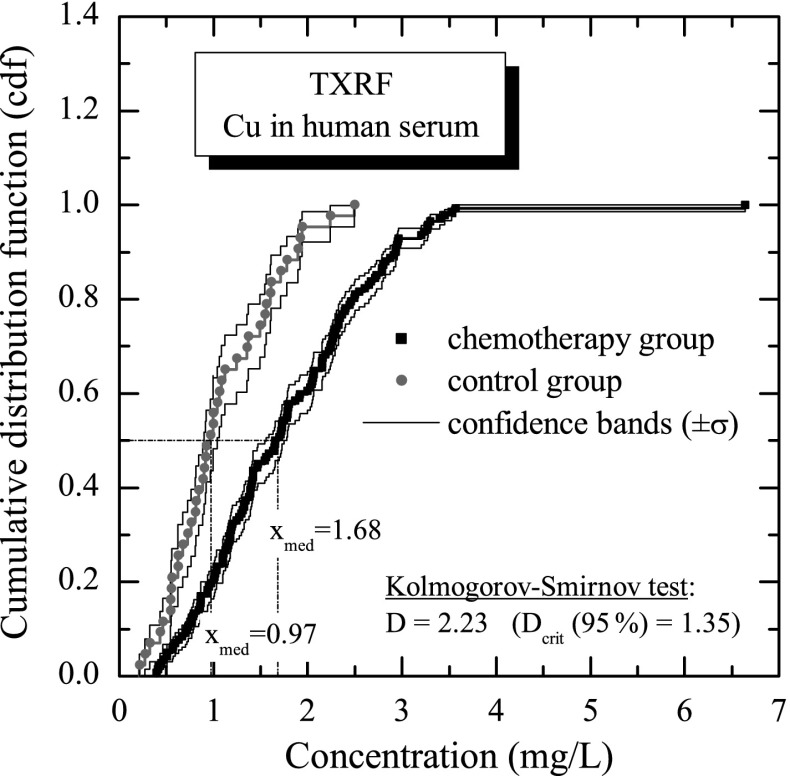
Comparison of cumulative distribution functions obtained (with ± *σ* confidence band) for copper concentrations of the control and chemotherapy groups, respectively. The figure shows the calculated median (*x*_med_) of the concentrations. The cumulative distribution functions were compared by using the nonparametric Kolmogorov-Smirnov test, which, on the confidence level of 95 %, confirms the statistical difference between copper concentration in human serum for the control and chemotherapy groups

In order to make final conclusions concerning on a possible difference between the level of copper concentration in two populations of samples, the control and chemotherapy groups have to be compared systematically. First of all, as it is shown in Fig. 6, the cumulative distribution functions (cdf) of copper concentration for both groups were calculated. Using the Greenwood formula [17] for the variance, the confidence bands of the empirical cumulative distribution function can be calculated for an assumed confidence level. In Fig. 6, the graphs of the empirical cumulative distribution functions are plotted together with curves of the confidence bands for the confidence level corresponding to the one standard deviation ( ± *σ*). The empirical cumulative distribution functions were statistically compared using the Kolmogorov-Smirnov *D* test [17], with the null hypothesis that the data in both populations are drawn from the same distribution. The Kolmogorov-Smirnov test does not require any particular assumption concerning on a form of the distribution. The calculated value of the test statistic (*D* = 2.23) for analysed group comparison exceeds the critical value (*D*_crit_ = 1.35). The final result of the test is a statistically significant difference between the level of copper concentration in the control group compared with the chemotherapy group, at the confidence level of 95 %. Knowledge on the empirical cumulative distribution function *F̂*(*x*) allows one to estimate the quantile *x̂*_*p*_ of *p* order (for example, median *x*_med_ ≡ *x̂*_0.5_) of the copper concentration distribution by solving the equation *F̂*(*x̂*_*p*_) = *p*. The *x̂*_*p*_ parameter is a value of the measured concentration for which *p* percent of measured concentration is less than *x̂*_*p*_. In statistical analysis, usually the following quantiles are discussed: *x̂*_0.05_, *x̂*_0.1_, *x̂*_0.25_, *x̂*_0.75_, *x̂*_0.9_, and *x̂*_0.95_. Basing on these quantiles, the corresponding ranges of the reference values can be defined: *x̂*_0.05_ ÷ *x̂*_0.95_ (5 % of the lowest and 5 % of the highest measured concentrations are rejected) and, respectively, the ranges *x̂*_0.1_ ÷ *x̂*_0.9_ and *x̂*_0.25_ ÷ *x̂*_0.75_. The values of the discussed quantiles and the reference ranges calculated in the presented studies for copper concentrations measured in control group are summarized in Table 2.

**Table 2:** The different ranges of the reference values of copper level in human serum obtained using the following quantiles calculated for the copper concentrations measured in the control group: *x̂*_0.05_, *x̂*_0.1_, *x̂*_0.25_, *x̂*_0.75_, *x̂*_0.9_, and *x̂*_0.95_

Control group		
Quantiles range (mg/L)		
*x̂*_0.25_ ÷ *x̂*_0.75_	*x̂*_0.1_ ÷ *x̂*_0.9_	*x̂*_0.05_ ÷ *x̂*_0.95_
0.625 ÷ 1.491	0.464 ÷ 1.90	0.273 ÷ 1.94

## Conclusions

The main aim of the presented work was concentrated on the determination of the copper concentration in the human serum samples for two different population, namely for the control group and for the chemotherapy patient group. The copper concentrations were determined in the samples by using the total reflection X-ray fluorescence method. This presented paper describes the main applications of this technique, the calibration procedure and the sample preparation procedure applied in the discussed studies. Performed measurements allowed the calculation of the copper concentration distribution parameters (mean value, standard deviation, median) for both analysed groups. The relatively large numbers of analysed samples for both studied groups give the possibility of presentation of the new methodology of data analysis. The advantage of this approach is the interpretation of the theoretical shape of the copper concentration distribution. It was found that the copper distribution in the control group is well described by the log-normal distribution, while in the chemotherapy group, the log-stable distribution was fitted. These observation were confirmed by the Anderson-Darling test. The log-stable characteristic of copper concentration distribution in serum of the chemotherapy group provides the appearance of extremely high copper concentration. The second new aspect was a statistical comparison of the copper concentration distributions for the studied group by using the Kolmogorov-Smirnov test with the conclusion that distributions are statistically different. The mean value and median of the copper level in chemotherapy patient group is much higher compared to that of the control group (1.08, 0.97 and 1.78 mg/L, and 1.68 mg/L, respectively). One of the main results of the performed studies was also the determination of copper concentration reference ranges in human serum based on the results obtained for the control group. Values of the mean, median and other quantiles determined in this case will be applied in our research programme, for example, in daily routine analysis of copper level in serum of patients with Hodgkin’s disease. The presented studies, which were concentrated on copper level which is of importance from the point of view of our research programme, we are going to extend by including the analysis of the concentration of other elements determined in human serum for chemotherapy and control groups as well as by discussion of the ratios of specific pairs of elements (Cu/Zn, Fe/Mn, Se/Cu). The number of the human samples in the control group will be also systematically increased in the near future. Additionally, in the studies on trace element concentration in serum of the chemotherapy patients, also the kind of the cancer and the stage of chemotherapy process will be taken into account. Parallel to the comparison of the levels of element concentration, also a discussion of the biochemical serum parameters will be performed in our next study.
